# Successful ablation of morphology-changing premature ventricular contractions under the guidance of local voltage potentials: A case report

**DOI:** 10.3389/fcvm.2022.1008380

**Published:** 2023-01-11

**Authors:** Yang Chen, Yuncai Zhu, Peng Zhang, Tao He, Jie Liao

**Affiliations:** ^1^Department of Cardiology, Sichuan Provincial People’s Hospital, University of Electronic Science and Technology of China, Chengdu, China; ^2^Department of Cardiology, Chinese Academy of Sciences Sichuan Translational Medicine Research Hospital, Chengdu, China; ^3^Department of Cardiology, Wenjiang District People’s Hospital of Chengdu, Chengdu, China

**Keywords:** premature ventricular contractions, QRS morphology, local voltage potentials, ablation, bi-morphology

## Abstract

Premature ventricular contractions (PVCs) stemming from the aortic sinus cusp often have preferential conduction to two exits in the outflow tract and exhibited two different morphologies of PVCs, which may render radiofrequency catheter ablation (RFCA) difficult. A 67-year-old male patient underwent RACF for premature ventricular contractions (PVCs) characterizing by bi-morphology (left and right bundle branch block) on electrocardiogram. Dynamic changes in QRS morphology during ablation and evident local voltage potentials during electro-anatomical mapping were critical for identifying the real foci of origin of PVCs. Successful ablation was achieved at the left-right coronary cusp commissure.

## Highlights

- Dynamic changes in QRS morphology of premature ventricular contractions (PVCs) during ablation and evident local voltage potentials during electro-anatomical mapping were critical for identifying the real foci of origin of PVCs.
- Premature ventricular contractions (PVCs) stemming from the left-right coronary cusp commissure often show preferential conduction to the RVOT.
- Bi-morphic QRS (LBBB and RBBB with an inferior axis) on ECG before the procedure may facilitate successful ablation of PVCs.

## Introduction

Premature ventricular contractions (PVCs) and ventricular tachycardia (VT) have plentiful origins, which are widely distributed in both the right and left ventricles, including outflow tract, the vicinity of the tricuspid/mitral annulus and the His bundle, the papillary muscle, and so on (1). PVCs usually have one exit showing a single QRS morphology, but sometimes have multiple exit sites showing polymorphic QRS in both the right ventricular outflow tract (RVOT) and left ventricular outflow tract (LVOT) with preferential pathways (2–6). Variation of PVCs origins and morphology, especially dynamic changes in QRS during ablation, is a challenge to location and ablation of PVCs (3, 4, 7). In addition, a local voltage potential (LVP) preceding the onset of the surface QRS during PVCs, may help identify the origin of PVCs, according to a previous study (8). Here, we report a case of PVCs, whose successful ablation depended on dynamic alterations in QRS morphology during ablation and evident local voltage potentials during electro-anatomical mapping, originating from the left-right coronary cusp commissure (L-RCC).

## Case report

**FIGURE 1:**
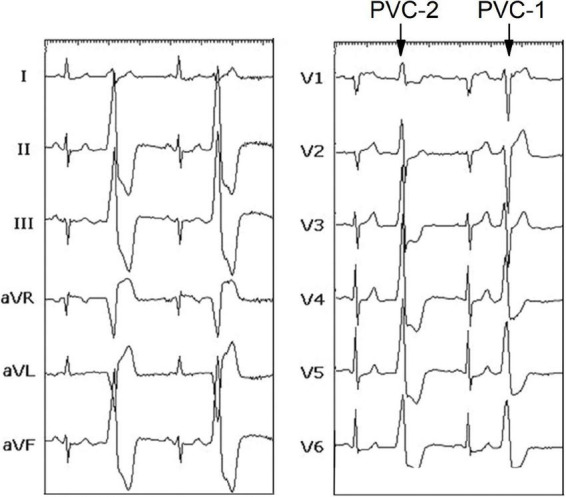
Twelve lead-ECG recorded before the procedure showing sinus rhythm, PVC-1, and PVC-2. ECG, electrocardiogram; PVC, premature ventricular contraction.

The patient was a 67-year-old man with a 2-year history of shortness of breath and frequent palpitation while resting. And he had a history of hypertension and coronary heart disease. The symptoms could not be explained by these diseases, because that LV ejection fraction (69%) was kept within reasonable bounds, and local stenosis in the middle left anterior descending coronary artery was unchanged compared to 2 years ago. In addition, electrocardiogram (ECG) was normal during sinus rhythm. However, a serious of ECGs show frequent bi-morphic PVCs characterized by left bundle branch block (LBBB) and/or right bundle branch block (RBBB) in Figure 1, whose QRS wave were upright in leads II, III, and aVF (an inferior axis). The PVCs with inverted (LBBB) and upright (RBBB) QRS wave in lead V1, were defined as PVC-1 and PVC-2, respectively. The coexistence of PVC-1 and PVC-2 was reconfirmed in the preoperative 24 h of ambulatory Holter monitoring, and PVC burden was 22,915 (26.3%), which was mainly composed of PVC-1. The symptomatic PVCs were refractory to antiarrhythmic medications. Therefore, this patient was referred for radiofrequency catheter ablation (RFCA) of the PVCs.

**FIGURE 2:**
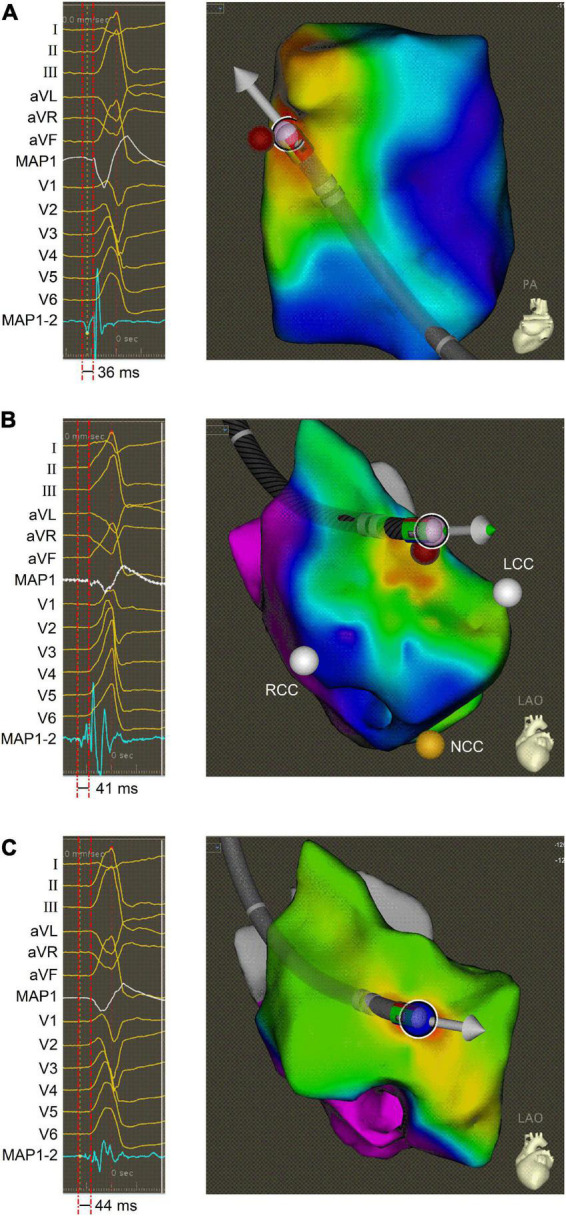
Activation mapping of PVCs. **(A)** Intracardiac ECG (**left** panel) and electro-anatomic map (**right** panel) obtained during activation mapping of PVC-1 in the anterior septum of RVOT. **(B)** Intracardiac ECG (**left** panel) and electro-anatomic map (**right** panel) obtained during activation mapping of PVC-2 near the LCC. **(C)** Intracardiac ECG (**left** panel) and electro-anatomic map (**right** panel) obtained during activation mapping of PVC-1 at the L-RCC. The bipolar ECG exhibited the LVP preceding the QRS onset, and electro-anatomic map showed ablation site in different electro-anatomic mapping areas. ECG, electrocardiogram; LCC, left coronary cusp; L-RCC, the left-right coronary cusp commissure; LVP, local voltage potential; MAP1, unipolar ECG; MAP1-2, bipolar ECG; RCC, right coronary cusp; PVC, premature ventricular contraction; RVOT, right ventricular outflow tract.

After informed written consent was given, the electrophysiologic study and RFCA for the PVCs were carried out using conventional fluoroscopically guided mapping, according to a previous report (3). The PVCs were featured with PVC-1 before the ablation procedure, suggesting that they originated from RVOT. Subsequently, electro-anatomic mapping (CARTO, Biosense Webster) of RVOT was performed using a 3.5-mm open irrigated tip (SmartTouch, Biosense Webster). The foci of origin of PVCs was determined using detailed activation and pace mapping. The site of earliest activation (−36 ms before the onset of the surface QRS), was distinguished, which was located in the anterior septum of RVOT (Figure 2A). Radiofrequency ablations (35 W, 17 ml/min, 2 min, 45°C) in this location reduced the occurrence of PVC-1, but the onset of PVC-2 shortly became frequent. Then, we performed electro-anatomical mapping in the aortic sinus cusp. The site of earliest activation (−41 ms before the onset of the surface QRS), was identified near the left coronary cusp (LCC) (Figure 2B). PVC-2 disappeared after similar ablation, but surprisingly, PVC-1 reappeared. However, Discrete and fragmented LVPs related to reappearing PVC-1 were observed near this ablation site, and then a systematical electro-anatomical mapping was again added to the aortic sinus cusp (ASC) based on previous research (3). The L-RCC was focused (−44 ms before the onset of the surface QRS) and PVC elimination was achieved after ablation in this location (Figure 2C). This patient recovered well and have been symptom-free for 3 months after discharge.

## Discussion and conclusion

Premature ventricular contractions (PVCs) and ventricular tachycardia (VT) originating from the ASC, which are typically regarded as originating from the LCC, the L-RCC, or the right coronary cusp (RCC), often show preferential pathway to RVOT, resulting in a LBBB morphology on ECG (3). In our case, we present PVCs with two morphologies (LBBB and RBBB with an inferior axis) on ECG, whose successful ablation was achieved at the L-RCC. Importantly, the QRS morphology surprisingly changed twice during ablation, and obvious LVPs were recorded during mapping, which were crucial to determination the real site of origin of PVCs.

Despite alteration in QRS morphology during ablation has been previously reported (4, 7), the reappearance of the QRS morphology (PVC-1) related to LBBB during ablation, was firstly described in the current case. Our findings further supported the hypothesis that PVCs originating from the ASC often show preferential conduction to the RVOT, exhibiting more LBBB and less RBBB on ECG. Moreover, the sequential shift from PVC-1 to PVC-2 to PVC-1, especially the reappearance of PVC-1, indicated that preferential conduction may be functional.

It is reported that LVPs are recorded close to the site of origin of ventricular ectopy in the vast majority of patients with idiopathic outflow tract ectopy, and LVPs may reflect an area of depressed conductivity known to be a prerequisite for experimental ventricular ectopy (8). Similarly, discrete and fragmented LVPs provided clues for the final successful ablation foci in this case. Of note, LVPs represents the area of impaired and anisotropic conduction, which may be an explanation for preferential conduction.

The process of ablation was not smooth due to misinterpretation of the PVCs origin. After several electro-anatomical mappings, attempted ablation was delivered sequentially at the RVOT, the site near the LCC, and the L-RCC. Luckly, successful ablation was achieved at the L-RCC. However, two QRS morphologies of PVCs (LBBB and RBBB with an inferior axis) on ECG, which may feature two breakout sites and only a single origin, could be a predictor of PVCs originating from ASC, according to a preceding report (3). Thus, bi-morphic QRS may have important implications for RACF in the current case. Specifically, on condition that the ablation at the RVOT was unsuccessful, electro-anatomical mapping should be added to the ASC without hesitation, which may make the operation effortless and efficient.

In the present case, our results have important clinical implications. Firstly, dynamic changes in QRS morphology of PVCs during ablation and evident LVPs during electro-anatomical mapping indicate PVCs may originate from the ASC. Secondly, bi-morphic QRS (LBBB and RBBB with an inferior axis) on ECG before the procedure may facilitate successful ablation of PVCs.

## Data availability statement

The raw data supporting the conclusions of this article will be made available by the authors, without undue reservation.

## Ethics statement

The studies involving human participants were reviewed and approved by the Ethics Committee of Sichuan Provincial People’s Hospital. The patients/participants provided their written informed consent to participate in this study. Written informed consent was obtained from the individual(s) for the publication of any potentially identifiable images or data included in this article.

## Author contributions

YC, YZ, and JL conceived the study. YC performed RACF. YZ collected ECG data and image data related to RACF. YC and YZ drafted the manuscript. JL, PZ, and TH checked the manuscript and performed critical revision. All authors approved the final version of the manuscript.
